# Exploring the β-tubulin gene family in a benzimidazole-resistant *Parascaris univalens* population

**DOI:** 10.1016/j.ijpddr.2021.08.004

**Published:** 2021-08-26

**Authors:** Frida Martin, Peter Halvarsson, Nicolas Delhomme, Johan Höglund, Eva Tydén

**Affiliations:** a Swedish University of Agricultural Sciences, Department of Biomedical Sciences and Veterinary Public Health, Section for Parasitology, Box 7036, 750 07, Uppsala, Sweden; b Umeå Plant Science Centre (UPSC), Department of Forest Genetics and Plant Physiology, Swedish University of Agricultural Sciences, 901 83, Umeå, Sweden

**Keywords:** Amplicon sequencing, Anthelmintic resistance, Equine, Fenbendazole

## Abstract

Benzimidazole (BZ) drugs are frequently used to treat infections with the equine ascarid *Parascaris univalens* due to increasing resistance to macrocyclic lactones and pyrantel. Benzimidazole resistance is rare in ascarids in contrast to strongyle parasites where this resistance is widespread. In strongyles, single nucleotide polymorphisms (SNPs) at codons 167, 198 and 200 in a β-tubulin gene have been correlated to BZ resistance, but little is known about the β-tubulin genes and their possible involvement in BZ resistance in *P. univalens* and other ascarids. Previously two β-tubulin genes have been identified in *P. univalens*. In this study, we present five additional β-tubulin genes as well as the phylogenetic relationship of all seven genes to β-tubulins of other clade III and V nematodes. In addition, the efficacy of fenbendazole for treatment of *P. univalens* on a Swedish stud farm was studied in 2019 and 2020 using faecal egg count reduction test. Reductions varied from 73% to 88%, indicating the presence of a resistant *P. univalens* population on the farm. The emergence of BZ resistance emphasizes the need for development of molecular markers for rapid and more sensitive detection of resistant populations. We therefore investigated whether possible SNPs at positions 167, 198 or 200 in any of the β-tubulin genes could be used to distinguish between resistant and susceptible *P. univalens* populations. Amplicon sequencing covering the mutation sites 167, 198 and 200 in all seven β-tubulin genes revealed an absence of SNPs in both resistant and susceptible populations, suggesting that the mechanism behind BZ resistance in ascarids is different from that in strongyle nematodes and the search for a molecular marker for BZ resistance in *P. univalens* needs to continue.

## Highlights

• First case of fenbendazole resistance in *Parascaris univalens* in Europe.
• The *P. univalens* β-tubulin family contains seven genes.
• *P. univalens* β-tubulin genes cluster with β-tubulins from other clade V nematodes.
• No resistance associated SNPs were identified in *P. univalens* β-tubulin genes.

## Graphical abstract

**Figure:**
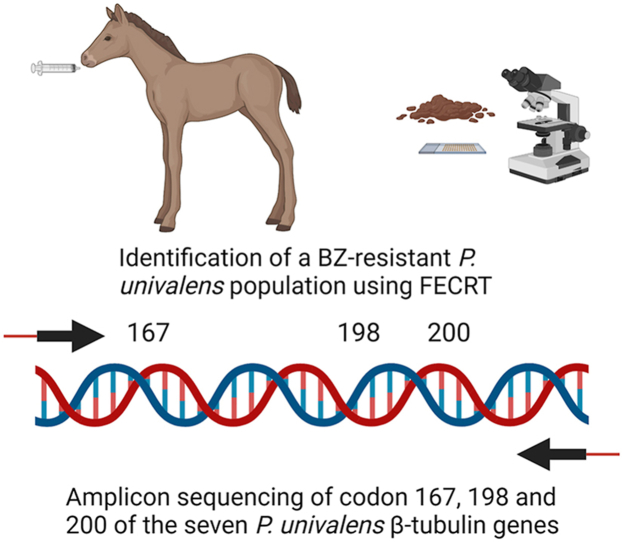

## 1 Introduction

The equine roundworm *Parascaris univalens* is a parasitic nematode that frequently infects foals and yearlings. Clinical signs include respiratory symptoms such as coughing and nasal discharge, lethargy, weight loss and impaired growth. In severe cases impaction and even rupture of the small intestine may occur and can be lethal (Clayton and Duncan, 1978; Cribb et al., 2006). Foals are usually treated regularly with anthelmintic drugs and anthelmintic resistance is therefore an increasing problem in *P. univalens* (Reinemeyer, 2009). Due to repeated reports of resistance to macrocyclic lactones (MLs) since 2002 (Boersema et al., 2002; Osterman Lind and Christensson, 2009; Reinemeyer, 2009) and more recently to pyrantel (PYR) (Lyons et al., 2008; Martin et al., 2018; Hautala et al., 2019), benzimidazoles (BZs) are currently the first choice of treatment for *P. univalens* infection in many countries. Despite frequent use of BZs, only a few cases of resistance have so far been reported in *P. univalens* (Armstrong et al., 2014; Alanazi et al., 2017). In *Ascaris lumbricoides* and *Ascaridia dissimilis*, the ascarid parasites of humans and turkeys, single cases of reduced efficacy have been noted (Collins et al., 2019; Krucken et al., 2017), while there have been no reported cases of resistance in the poultry and pig roundworms *Ascaridia galli* and *Ascaris suum* (Tarbiat et al., 2017; Zhao et al., 2017). This is in contrast to strongyle nematodes where resistance to BZ drugs appeared soon after introduction of the first BZ on the market and are now considered the most widespread type of anthelmintic resistance (Conway, 1964; Saunders et al., 2013).

Benzimidazole drugs act by binding to β-tubulin molecules, causing a conformational change and thereby hindering the polymerization of α- and β-tubulins in the construction of microtubules and other essential cellular components. This results in disruption of several essential functions of the cell, leading to starvation and death of the parasite (Lacey, 1990). Most nematodes have several β-tubulin genes, usually referred to as isotypes, and there is compelling evidence that single nucleotide polymorphisms (SNPs) in the β-tubulin isotype 1 gene are involved in BZ resistance in strongyle nematodes (von Samson-Himmelstjerna et al., 2007). In the sheep parasite *Haemonchus contortus* a SNP resulting in amino acid substitution from phenylalanine to tyrosine at position 200 was first identified and linked to BZ resistance and is believed to be the principal cause of BZ resistance in this species (Kwa et al., 1994). However, SNPs causing amino acid substitutions from phenylalanine to tyrosine at position 167 and from glutamate to alanine or leucine at position 198 have also been correlated to BZ resistant phenotypes (Silvestre and Cabaret, 2002; Ghisi et al., 2007; Redman et al., 2015; Avramenko et al., 2019). In addition, it has been suggested that mutations in the isotype 1 gene are not solely responsible, but that BZ resistance may be multi-genic. For example, SNPs in the *H. contortus* β-tubulin isotype 2 gene have been correlated to BZ resistance in field strains (Kwa et al., 1993a, 1993b) and BZ drugs have also been shown to influence the expression of drug metabolizing enzymes and drug efflux pumps (Beugnet et al., 1997; Laing et al., 2010; Jones et al., 2013; Kellerova et al., 2020).

Although it is generally accepted that the main molecular target for BZ anthelmintics in nematodes is β-tubulin, the knowledge of the diversity of the tubulin family has increased in recent years, indicating that the level of complexity is higher than previously thought (Kotze et al., 2014). For example, the model organism *Caenorhabditis elegans* has six β-tubulin genes (Gogonea et al., 1999), though only one of these, *ben-1*, is a drug target for BZs (Driscoll et al., 1989). Several mutations in this gene have been reported to confer BZ resistance, including the polymorphisms described above in the β-tubulin isotype 1 gene (Driscoll et al., 1989; Dilks et al., 2020). Saunders et al. (2013) presented a thorough investigation of the β-tubulin family of *H. contortus* and found that it consists of four isotypes and that isotypes 1 and 2 have a paralogous relationship to *C. elegans ben-1*, *tbb-1* and *tbb-2*. In *Parascaris* spp. two β-tubulin genes have been identified so far, at the time named isotype 1 and isotype 2 (Tyden et al., 2013). However, since these showed phylogenetic diversity compared to β-tubulin genes of nematodes of other genus these names may be misleading and will not be used further in this paper. In the related poultry roundworm *A. galli* six β-tubulin genes have been identified (Martis et al., 2017).

The possible presence of resistance associated SNPs in β-tubulin genes have also been investigated in ascarid worms. None of the previously described polymorphisms were found in either of the two β-tubulin genes in BZ-susceptible *Parascaris* spp. (Tyden et al., 2013, 2014) and similarly Tarbiat et al. (2017) found no SNPs in a β-tubulin gene of BZ-susceptible *A. galli*. In *A. lumbricoides*, no SNPs could be detected at any of the sites of four β-tubulin genes in a population with a reduced efficacy of the BZ albendazole (Krucken et al., 2017). However, Diawara et al. (2009) found a polymorphism at position 167 in a β-tubulin gene of an albendazole-susceptible population and Furtado et al. (2019) found a mutation at codon 200 at 0.5% allele frequency in a population with unknown resistance status.

There have been several efforts to develop methods to use SNPs in the β-tubulin isotype 1 gene as molecular markers for detection of anthelmintic resistance, using techniques such as quantitative PCR, pyrosequencing, droplet digital PCR and amplicon sequencing (Alvarez-Sanchez et al., 2005; von Samson-Himmelstjerna, 2006; Baltrusis et al., 2018; Avramenko et al., 2019). A molecular maker for BZ resistance in *P. univalens* would allow easy and rapid screening of foal faecal samples to identify resistance to BZ, rather than the more laborious Faecal Egg Count Reduction Test (FECRT). In addition, since resistance can only be detected by FECRT when the proportion of the parasite population carrying the resistance gene exceeds 25%, a molecular marker would make earlier detection of resistance possible compared to using FECRT (Martin et al., 1989).

In this study we present the seven genes in the *P. univalens* β-tubulin family and their phylogenetic relationship to β-tubulin genes of other clade III and V nematodes. We have identified a population of BZ-resistant *P. univalens* at a Swedish stud farm using FECRT and used this unique population to search for previously described SNPs at codons 167, 198 and 200 in the seven β-tubulin genes.

## 2 Materials and methods

### 2.1 Farms, foals and collection of parasite material

Research ethics committee oversight was not required for this research according to the Swedish National Board of Agriculture (SJVFS, 2019:9, L159). Written owner informed consent was obtained for all included animals.

In total 63 foals from two farms (farm 1 and farm 2) were included in FECRTs and used to collect parasite eggs for amplicon sequencing. Data on the included farms and foals are shown in Table 1.

**Table 1:** Data on farms included in faecal egg count reduction test and amplicon sequencing.

Farm	Group	Year	Group size	Age (months)	Group mean EPG pre^a^	Group mean EPG post^a^	Max individual EPG pre^a^	Max individual EPG post^a^	FECR (%)	% of foals excreting ≥50 EPG^b^	% of foals with increasing EPG^c^
1	A	2019	16	5–9	560	125	1500	800	78	38	6
1	B	2020	35	3–6	1939	523	7350	3000	73	74	23
1	C	2020	11	3–6	1968	232	5200	1600	88	36	0
2	D	2020	4	5–6	1725	0	2800	0	100	0	0

a Pre- and post-treatment egg counts.

b Percentage of foals excreting 50 EPG or more post-treatment.

c Percentage of foals with higher EPG post treatment than pre-treatment.

Farm 1 monitor egg counts for *P. univalens* at monthly intervals and treat foals with ≥100 Eggs Per Gram faeces (EPG). Fenbendazole (FBZ) has been used to treat the foals on the farm for approximately 15 years. A FECRT was performed with foals on the farm in two consecutive years. In 2019, 16 foals between five and nine months of age were included (group A). In 2020, 35 foals (group B) and 11 foals (group C) between three and six months of age were included. Eggs excreted after treatment with FBZ were collected from all groups for amplicon sequencing.

Farm 2 has a documented 100% efficacy of FBZ (Martin et al., 2018) and was included to serve as a control for the amplicon sequencing. A FECRT was performed in 2020 and included four foals between five and six months of age (group D). Eggs from all included foals were collected pre-treatment and used for amplicon sequencing.

### 2.2 Faecal Egg count reduction test

Individual faecal samples were collected from the foals described above, placed in double plastic bags and posted to our laboratory (Section for Parasitology, Swedish University of Agricultural Sciences). Paired faecal egg counts were performed pre- (day 0) and post-treatment (day 11–20) using a modified McMaster technique with a minimum detection limit of 50 EPG (Coles et al., 1992). All foals excreted ≥100 EPG and were treated with FBZ paste (Axilur® MSD, Kenilworth, USA) at a dosage of 7.5 mg/kg as recommended by the manufacturer. All foals were weighed or measured to estimate the weight and the anthelmintic drug was dosed for additional 50 kg to avoid under dosing. Faecal sampling, weighing and treatment were performed by experienced stud personnel. The faecal egg count reduction was calculated on paired samples by a Bayesian hierarchical model on the shiny-eggCounts web interface (Torgerson et al., 2014). The results were interpreted according to suggested cutoff values for strongyle nematodes by the American Association of Equine Practitioners (AAEP) (Nielsen et al., 2019) as there are currently no specific guidelines for *P. univalens* FECRT.

### 2.3 Examination of the β-tubulin family

#### 2.3.1 Identification of β-tubulin genes in the *P. univalens* genome

β-tubulin genes were identified from the functional annotation of RNA-Seq data (PRJEB37010) (Martin et al., 2020). Manual BLAST searches in the *P. univalens* genome (PRJNA386823) (Wang et al., 2017) in WormBase Parasite (Howe et al., 2017) using the RNA-Seq genes as template did not identify any additional β-tubulin genes. All identified sequences were confirmed to contain a β-tubulin chain by BLASTP searches in the NCBI database (Sayers et al., 2021).

#### 2.3.2 DNA extraction

Eggs were isolated and decorticated according to Martin et al. (2018). For each group (A, B, C and D) approximately 30,000 eggs were incubated 14 days at 28 °C for development into larvae, then genomic DNA was prepared in two separate reactions for each group, using NucleoSpin® Tissue kit (Macherey Nagel, Düren, Germany) according to manufacturer's instructions. DNA concentration was determined using Qubit BRDNA kit (Invitrogen, Carlsbad, USA).

#### 2.3.3 Primer design and validation

Primer3 web (Untergasser et al., 2012) was used to design primers for amplicons containing SNP sites 167, 198 and 200 in each of the seven β-tubulin genes (Table 2). Due to differences in intron length between the β-tubulin genes, the expected amplicon size ranged from 559 to 1834 bp. Unique barcodes to identify genes and groups were added to the primers. Primers of NGS quality were ordered from Eurofins Genomics (Galten, Denmark). To confirm primer specificity PCR reactions containing: 12.5 μl Accustart II PCR ToughMix (Quanta bio, Beverly, USA), 1 μl F Primer (10 μM), 1 μl R primer (10 μM), 9.5 μl H_2_O and 1 ng template DNA were prepared and performed at 94 °C for 3 min, followed by 35 cycles of 94 °C for 30 s, 58 °C for 30 s and 72 °C for 90 s with a final extension at 72 °C for 9 min. PCR products were confirmed by gel electrophoresis, Sanger sequencing (Macrogen Europe, Amsterdam, Netherlands) and alignments in MUSCLE Multiple Sequence Alignment (Madeira et al., 2019).

**Table 2:** *Parascaris univalens* β-tubulin genes, primers used for sequencing and amplicon sizes.

Putative gene name	WormBase Parasite ID	Amplicon sequencing primers	Amplicon size (bp)
*tbb-3*	PgB04_g135	F: GCGAGCGTATGGAAAGAGAG R: AGCAAAGAGCTGATGGTCGT	1834
*tbb-4*^a^	PgB04_g136	F: TCGGAATCCATAAGCTCTGC R: CTTGCGATTTGATTGGAACA	559
*tbb-5*^b^	PgR007_g022	F: TGTGAGAAAATGCCATCGTG R: GCAACCTCCATCCGAATACT	685
*tbb-6*^c^	PgE153_g002	F: ATTGCTGAAGGTTTGGTTCG R: CTCCGATTTCGTTGCTTCTC	1246
*tbb-7*	PgB10_g062	F: CAACAAAGCTCGTTTTGAAGG R: TTAATGCAACGACGTTCCTG	997
*tbb-8*	PgR003_g161	F: CACGCTTCGCTCTTCTTAGG R: ATCGGCACAAAAATGGAAAC	840
*tbb-9*	PgR045_g070	F: TTCAAACAACGGCGATGATA R: AATTTCACATCTCCGGATGC	971

a Incomplete readingframe in WormBase Parasite.

b Previously described as β-tubulin isotype 1 (KC713797).

c Previously described as β-tubulin isotype 2 (KC713798).

#### 2.3.4 Amplicon generation and sequencing

To minimize PCR bias two reactions were performed for each β-tubulin gene. Amplicons were generated using 30 μl Phusion Hot Start II High-Fidelity PCR Master Mix (Thermo Fisher Scientific, Waltham, USA), 3 μl of each specific barcoded primer (10 μM), 15 ng of specific DNA and molecular grade water (Qiagen, Hilden, Germany) up to 60 μl. PCR reactions were run on Applied Biosystems (Foster City, USA) 2721 Thermal Cycler for 1 min at 98 °C followed by 24 cycles of 10 s at 98 °C and 45 s at 72 °C with a final extension for 10 min at 72 °C. Amplicons were purified using AMPure XP magnetic beads (Beckman Coulter Inc., Brea, USA) according to the manufacturer's instructions (0.8 x). DNA concentration was determined using Qubit BRDNA kit. To get a similar number of amplicons for each gene in the multiplex library the amount of each amplicon in the final library was calculated based on amplicon size and concentration and then pooled.

After quality control using TapeStation (Agilent, Santa Clara, USA), 500 ng of total amplicon metabarcoded pool was sequenced on a SMRT cell (Pacific Biosciences, Meleno Park, USA) using the PacBio Sequel Technology Platform (Uppsala Genome Center (UGC), Science for Life Laboratory, Dept. of Immunology, Genetics and Pathology, Uppsala University, BMC, Box 815, SE-752 37 UPPSALA).

#### 2.3.5 NGS data analysis

Initial raw PacBio sub-read datasets were processed to generate unaligned Circular Consensus Sequence (CCS) reads with the SMRT Link v9.0 (Pacific Biosciences) software pipeline using CCS application with default parameters. The CCS reads from all samples were mapped independently to the genes of interest in the *P. univalens* reference genome (PRJNA386823) with the SMRT pbmm2 tool kit (Pacific Biosciences). Demultiplexing was performed on aligned CCS reads to retrieve independent sample reads. Variant calling (SNP and Indels) was performed with GATK4v4 HaplotypeCaller (DePristo et al., 2011) and multi-sample variant calling was performed for each group (A, B, C and D) using CombineGVCFs and GenotypeGVCFs functions.

#### 2.3.6 De novo assembly of β-tubulin genes

To extend the incomplete coding sequence of PgB04_g136 available in WormBase Parasite (Howe et al., 2017) RNA-Seq data (PRJEB37010) from Martin et al. (2020) was used for a *de novo* transcriptome assembly using Trinity version 2.11 (Grabherr et al., 2011) including the PacBio β-tubulin reads as extra support. The transcriptome was turned into a BLAST database using blast + version 2.6.0+ (Camacho et al., 2009). *Parascaris univalens* β-tubulin mRNA sequences were retrieved from WormBase Parasite (Howe et al., 2017) release 15, Oct 2020 and aligned against the transcriptome database to identify β-tubulin transcripts. The results were processed by wrapping up code published in the UPSCb-common repository (https://doi.org/10.5281/zenodo.4001774), as described in this manuscripts companion code repository (https://github.com/SLUBioinformaticsInfrastructure/parascaris-project).

#### 2.3.7 Phylogenetic analysis

Sequences homologous to *P. univalens* β-tubulin genes were retrieved from WormBase Parasite (Howe et al., 2017) and the protein sequences were obtained using Transdecoder v5.5.0 (https://github.com/TransDecoder/TransDecoder). β-tubulin sequences for clade III and V parasites *A. lumbricoides*, *A. suum*, *Toxocara canis, Brugia malayi*, *H. contortus*, *Necator americanus* and *Onchocerca volvulus* were included provided they had a reciprocal percentage identity over 60% and a full-length coding sequence in WormBase Parasite (Howe et al., 2017). Sequences for the complete *C. elegans* β-tubulin family were retrieved from WormBase (Harris et al., 2020) version WS280, however *Cel-tbb*-6 was excluded from the analysis to avoid long branch attraction (Saunders et al., 2013) (Table 3). *Saccharomyces cerevisiae* β-tubulin mRNA (NP_116616.1) was used as outgroup.

**Table 3:**
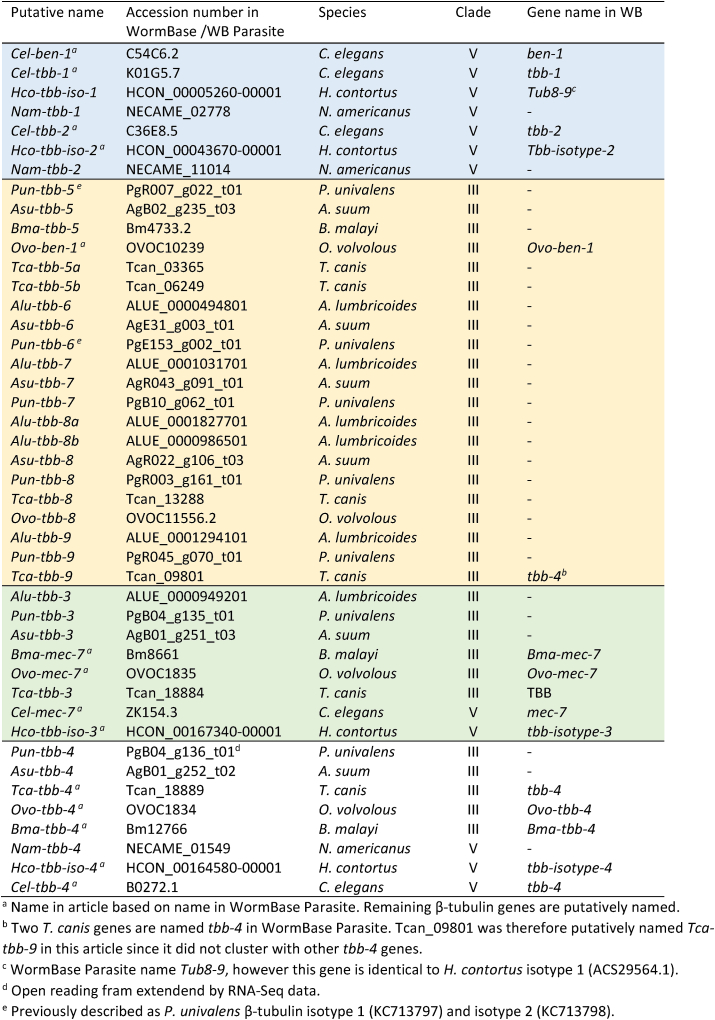
Sequences used for phylogenetic analysis, blue indicates a clade V β-tubulin cluster, orange a cluster containing clade III β-tubulins. Isotype-3 cluster (green) and isotype-4 cluster (white) contain β-tubulins from both clade III and V.

The phylogenetic analysis was performed on the Phylogeny.fr platform, advanced mode, using default settings (Dereeper et al., 2008, 2010). Sequences were aligned using MUSCLE (v3.7), then regions containing gaps or poorly aligned sequence were removed with Gblocks (v0.91b) using the less stringent selection (Castresana, 2000; Edgar, 2004). The phylogenetic relationship between the sequences was analysed using the maximum likelihood method implemented in PhyML (v3.0) using the default substitution model (Guindon and Gascuel, 2003; Anisimova and Gascuel, 2006). Graphical representation and editing of the phylogenetic tree were performed with TreeDyn (v198.3) (Chevenet et al., 2006). Finally, the R package ggtree was used to visualise the reconstructed tree (Yu, 2020).

## 3 Results

### 3.1 Faecal egg count reduction test

Group A, B and C on farm 1 had observed efficacies of 78%, 73% and 88% respectively (Table 1), which is below the resistance threshold of 90% suggested by the AAEP (Nielsen et al., 2019). The proportion of foals shedding eggs at the post-treatment sampling was 38% in group A, 74% in group B and 36% in group C (Table 1). In addition, 6% of the foals in group A and 23% of the foals in group B shed more eggs post-treatment than pre-treatment. In group D on farm 2 the observed efficacy was 100% (Table 1).

### 3.2 Identification of β-tubulin genes and phylogenetic analysis

Seven β-tubulin genes were identified based on functional annotation of RNA-Seq data (PRJEB37010) from one of our previous projects (Table 2). Two of these, PgR007_g022 and PgE153_g002 corresponded to the previously described β-tubulin genes (Tyden et al., 2013) while the remaining five were novel β-tubulin genes. All seven genes were confirmed β-tubulins by BLASTP searches in the NCBI database (Sayers et al., 2021). All β-tubulin genes had complete reading frames in WormBase Parasite apart from PgB04_g136, which was extended to contain the full coding sequence by a *de novo* assembly using RNA-Seq data as above combined with PacBio data from the amplicon sequencing.

**Fig. 1:**
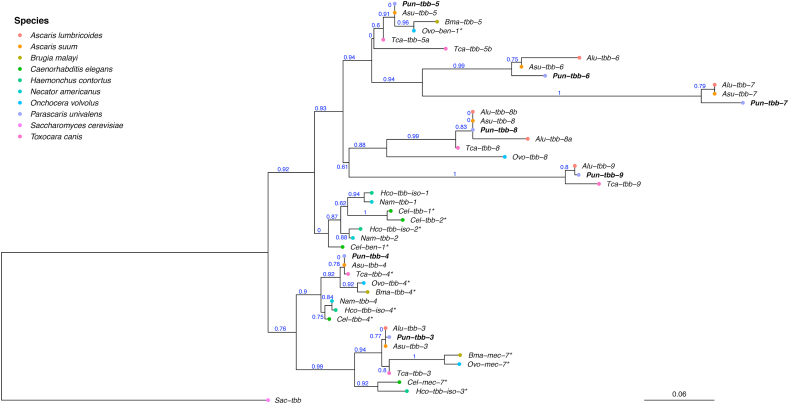
**Phylogenetic tree of Β-tubulin genes**. The tree was constructed on the Phylogeny.fr platform, using a maximum likelihood based analysis of seven *Parascaris univalens* Β-tubulin genes and their full length homologues in *Ascaris lumbricoides*, *Ascaris suum*, *Toxocara canis, Brugia malayi*, *Haemonchus contortus*, *Necator americanus* and *Onchocerca volvulus* retrieved from WormBase Parasite and *Caenorhabditis elegans* obtained from WormBase. *Saccharomyces cerevisiae* was used as outgroup for the phylogenetic analysis. * Gene name from WormBase, all other gene names are putative (Table 3).

The phylogeny of the seven β-tubulin genes from *P. univalens* were compared to full length β-tubulin genes from eight other nematodes belonging to clade III and clade V and formed four major clusters (Fig. 1, Table 3). Genes from both clades formed two different clusters containing isotypes 3 and 4. PgB04_g135 was putatively named *Pun-tbb-3* since it clustered with *Hco*-*tbb-iso-3* and *C. elegans mec-7*. Genes from all species included in the analysis were present in this tbb-3-cluster, apart from *N. americanus,* possibly due to the lack of a full length sequence in WormBase Parasite for this gene. Similarly, PgB04_g136 was putatively named *Pun*-*tbb-4* since it formed a cluster with *Hco*-*tbb-iso-4* and *C. elegans tbb-4*. In this tbb-4-cluster, genes from all included species except *A. lumbricoides* were present, again possibly due to the lack of a full length sequence in WormBase Parasite. In addition, two separate clusters were formed by clade III and clade V β-tubulin genes. In the clade V-cluster *H. contortus* isotype 1 and isotype 2 as well as *C. elegans ben-1*, *Cel-tbb-1* and *Cel-tbb-2* grouped together. In a separate cluster the two previously described β-tubulin genes PgR007_g022 and PgE153_g002 as well as the novel PgB10_g062, PgR003_g161 and PgR045_g070, putatively named *Pun-tbb-5, Pun-tbb-6, Pun-tbb-7*, *Pun-tbb-8* and *Pun-tbb-9*, grouped with previously unnamed β-tubulin genes from clade III nematodes including ascarids (Fig. 1, Table 3).

### 3.3 Amplicon sequencing

All seven β-tubulin genes of *P. univalens* were examined for the presence of resistance associated SNPs at position 167, 198 and 200 in samples from three groups with reduced efficacy of FBZ (farm 1 - group A, B and C) and one group with 100% efficacy of FBZ (farm 2 - group D). PacBio sequencing yielded between 1741 and 20,611 consensus reads for each amplicon.

No sequence polymorphisms were identified in any of the examined sites when compared between farms with resistant phenotype, farms with susceptible phenotype and the *P. univalens* reference genome from WormBase Parasite. However, *Pun-tbb-7* (PgB10_g062) contained a tyrosine at position 200 in samples from all groups as well as in the reference genome.

## 4 Discussion

This is the first report of FBZ resistance in *P. univalens* in Sweden and Europe. Since resistance to MLs is considered to be widespread in Sweden (Lindgren et al., 2008; Osterman Lind and Christensson, 2009) and resistance to PYR has been found on a number of farms (Martin et al., 2018), these results imply that multi-resistant *P. univalens* populations may be present on Swedish stud farms. This increasing problem with anthelmintic resistance in *P. univalens* is a major threat to equine health and welfare, but also to the equine industry. The efficacy of FBZ has been monitored regularly in Sweden for the past 12 years, with results of 100% (Osterman Lind and Christensson, 2009; Tyden et al., 2013) until 2018 when one farm was classed as resistant (Martin et al., 2018). However, this was due to treatment failure of only two out of 13 foals and therefore not considered as a clear case of FBZ-resistance at the time (Martin et al., 2018). In our current study, the same farm (farm 1) showed efficacies below the 90% resistance threshold (Nielsen et al., 2019) in both 2019 and 2020 and these results were further supported by the fact that a large proportion of the foals excreted eggs post treatment. Resistance to BZ-drugs is rare in *Parascaris* spp. and has so far only been demonstrated in Australia (Armstrong et al., 2014) and Saudi Arabia (Alanazi et al., 2017). Therefore, this is a unique population for exploration of mechanisms behind BZ resistance in *P. univalens*.

The rapid emergence of BZ resistance suggests that regular monitoring of drug efficacy is important, especially in *P. univalens*, since treatments are usually performed routinely without previous faecal examination. Since it has been suggested that FECRT only detect resistance when more than 25% of the population carries the resistance phenotype (Martin et al., 1989), development of alternative methods for early detection of resistance, such as molecular markers, is important.

It is therefore valuable to explore the β-tubulin family of *P. univalens* and examine the genes for SNPs associated with resistance in other nematodes. Using RNA-Seq data from *P. univalens*, we found that the β-tubulin family contains seven genes. We also investigated the phylogenetic relationship of these genes with β-tubulin genes from the model organism *C. elegans* and parasites of importance in clades III and V. Two β-tubulin genes have previously been described in *P. univalens*, and were then named isotype 1 and isotype 2 (Tyden et al., 2013). However, since it is now known that these genes are not orthologous to the strongyle β-tubulin isotype 1 and isotype 2 genes the names are misleading and we have therefore renamed them *Pun-tbb-5* and *Pun-tbb-6* in this article. The phylogenetic analysis show that the *P. univalens* β-tubulin genes *Pun-tbb-5*, *Pun-tbb-6 Pun-tbb-7*, *Pun-tbb-8* and *Pun-tbb-9* are situated in a clade together with other ascarid β-tubulins but separated from and equally related to *Cel-ben-1*, *Cel-tbb-1* and *Cel-tbb-2* as well as *Hco-tbb-iso-1* and *Hco-tbb-iso-2*. Functional studies in *C. elegans* has shown that *Cel-ben-1* is the only established drug target of BZs (Driscoll et al., 1989) and in *H. contortus* several studies support the involvement of *Hco-tbb-iso-1* and also suggest that *Hco-tbb-iso-2* is involved in BZ resistance (Kwa et al., 1993a; Rufener et al., 2009). Even though *Cel-tbb-1* and *Cel-tbb-2* are closely related to *Cel-ben-1*, they are suggested not to be targets for BZs as they carry a tyrosine in position 200, rather than the phenylalanine needed for BZ binding (Saunders et al., 2013). According to our results, the *P. univalens* β-tubulin genes *Pun-tbb-3* and *Pun-tbb-4* are closely related to *Hco-tbb-iso-3* and *Cel-mec-7* as well as to *Hco-tbb-iso-4* and *Cel-tbb-4* respectively (Fig. 1). However, *Cel-mec-7* and *Cel-tbb-4* are only expressed in specific neurons and are therefore not considered to be involved in BZ binding or resistance (Savage et al., 1989; Hurd et al., 2010). Saunders et al. (2013) concluded that the same is true for *Hco-tbb-iso-3* and *Hco-tbb-iso-4*, and we therefore suggest a similar function for *Pun-tbb-3* and *Pun-tbb-4*. We found that *Pun-tbb-7* carry a tyrosine at position 200 in samples from both resistant and susceptible phenotypes, as well as in the reference genome derived from a population of anthelmintic naïve *P. univalens* (Wang et al., 2017). Based on these results and the specific expression and relationship of isotype-3 and isotype-4 genes discussed above, our data suggest that *Pun-tbb-3*, *Pun-tbb-4* and *Pun-tbb-7* are most likely not targets for BZ-drugs in *P. univalens*. However, one or several of the genes *Pun-tbb-5*, *Pun-tbb-6*, *Pun-tbb-8*, or *Pun-tbb-9* could be candidates for BZ drug-target and resistance in *P. univalens* as they are equally related to *Cel-ben-1* and express a phenylalanine at position 200.

We sequenced amplicons covering the mutation sites 167, 198 and 200 in the seven β-tubulin genes from three pools of *P. univalens* larvae with a FBZ-resistant phenotype and one pool of larvae with a FBZ-susceptible phenotype. No SNPs at the positions responsible for BZ resistance in strongyles were identified in any of the seven β-tubulin genes, neither in the FBZ-resistant or susceptible phenotypes. These results are in line with results from Krucken et al. (2017) who found no resistance associated mutations in *A. lumbricoides* showing reduced efficacy of BZs. Since none of the SNPs associated with resistance in strongyle nematodes were identified in FBZ-resistant *P. univalens* the search for BZ resistance mechanism continues. Since as many as 28 mutations in the *C. elegans ben-1* gene were identified in connection to BZ resistance (Driscoll et al., 1989) there is a possibility that a mutation in another position could be responsible for BZ resistance in *P. univalens*. Any breakthroughs in this area would also be important in a one health aspect as *P. univalens* are closely related to *A. lumbricoides* that infects 819 million people yearly, mainly children (Pullan et al., 2014). Since this parasite is treated by mass drug administration and reduced efficacy of BZ drugs has been noted (Krucken et al., 2017) there is an urgent need for molecular markers to identify resistance also in ascarids (Prichard, 2007).

## 5 Conclusions

This is the first report of BZ-resistance in an ascarid parasite in Europe and the first presentation of the full β-tubulin family of an ascarid parasite. These results are of major importance for further exploration of BZ resistance in *P*. *univalens*, and possibly also in the related human ascarid parasite *A. lumbricoides*.

Phylogenetic analysis indicates that *Pun-tbb-5*, *Pun*-*tbb-6*, *Pun-tbb-8*, or *Pun-tbb-9* could be candidates for BZ drug interaction and resistance in *P. univalens*, however more research is required to confirm this. Amplicon sequencing of the sites for resistance associated SNPs in strongyle nematodes, 167, 198 and 200, did not identify any polymorphisms in any of the *P. univalens* β-tubulin genes in samples from a farm with reduced efficacy to FBZ. This suggests that the mechanisms behind BZ resistance in ascarids are different than in strongyle nematodes.

The rapid emergence of BZ resistance in *P. univalens* suggests that regular monitoring of drug efficacy is important since treatments are usually performed routinely without previous examination of faecal samples. Since resistance to all registered anthelmintic drugs have been identified in *P. univalens* it is also of great importance to improve biosecurity and hygiene measures on stud farms to lower infection pressure and spread of resistant parasites.

## Funding

This work was supported by the 10.13039/501100001862Swedish research council FORMAS (grant number 942-2015-508) and the Swedish-Norwegian Foundation for Equine Research (grant number H-20-47-556).

## Data availability

The PacBio CCS data has been deposited at the European Nucleotide Archive (https://www.ebi.ac.uk/ena/browser/home) under the accession number PRJEB47205.

## Code availability

All the code used for the analyses (apart from those conducted on Phylogeny.fr) are available from the GitHub repository: https://github.com/SLUBioinformaticsInfrastructure/parascaris-project.git.

## Declaration of competing interest

The authors declare that they have no known competing financial interests or personal relationships that could have appeared to influence the work reported in this paper.
